# Dihydroorotase from *Methanococcus jannaschii* with substrate bound

**DOI:** 10.1063/4.0001171

**Published:** 2025-10-27

**Authors:** Jacqueline Vitali, Jay C. Nix, Haley E. Newman, Michael J. Colaneri

**Affiliations:** 1 Cleveland State University, Cleveland, OH, USA; 2 Lawrence Berkeley National Laboratory, Berkeley, CA, USA; 3 SUNY at Old Westbury, Old Westbury, NY, USA

## Abstract

Dihydroorotases (DHOase) catalyze the reversible cyclization of N-carbamoyl-L-aspartate (CA) to L-dihydroorotate (DHO) in the third step of de novo pyrimidine biosynthesis. Here we report the x-ray structural analysis of DHOase from Methanococcus jannaschii co-crystallized with DHO at pH 6.5. The crystals are isomorphous with the crystals of the apoenzyme (Vitali et al, 2023) with space group P3221 and a = b = 111.4 Å, c = 101.2 Å. The structure was refined to R = 0.169 and Rfree = 0.186 at a resolution of 1.87 Å. The electron density in the active site corresponds to the substrate (CA), indicating that during crystallization the degradative reaction took place and DHO was converted to CA. The flexible loop (residues 140-151) displays two alternate, conformations. One conformation is in the loop-out form and is very similar to the flexible loop observed in the apoenzyme structure (Vitali et al, 2023). The second conformation is in the loop-in form and has weaker electron density. The interactions of the CA with the enzyme, shown in Fig. 1, involve the side chains of invariant residues R60, H306, N89, D302 and the main chain of S320 and N275 that correspond to invariant positions. In addition, CA interacts with the two Zn ions in the active site. Finally, CA interacts with the side chain of S143 and V144 of the loop-in form of the flexible loop. These interactions are similar to E. coli (Lee et al, 2007) and human (Grande-Garcia et al, 2014) DHOases, the largest differences being in the interactions with the flexible loop. In E. coli (Lee et al, 2007) and human (Grande-Garcia et al, 2014) DHOases, during catalysis, the flexible loop closes as a lid over the active site stabilizing the substrate and the transition state. This is the first time that the flexible loop is mostly in the loop-out form in the presence of the substrate CA, challenging the traditional view.
